# Prognosis and Therapies in Non-Clear Cell Renal Cell Carcinomas

**DOI:** 10.3390/cancers18193224

**Published:** 2026-10-07

**Authors:** Trilok Shrivastava, Yusra Medik, Nikhila Sampath Kumar, Shi-Ming Tu, Anuradha Kunthur

**Affiliations:** 1 Department of Hematology & Oncology, Winthrop P. Rockefeller Cancer Institute, University of Arkansas for Medical Sciences, Little Rock, AR 72002, USA; tshrivastava@uams.edu (T.S.); ymedik@uams.edu (Y.M.); nsampathkumar@uams.edu (N.S.K.); 2 Department of Hematology & Oncology, Renown Health, Reno, NV 89502, USA; shiming.tu@renown.org

**Keywords:** non-clear cell renal cell carcinoma, papillary renal cell carcinoma, chromophobe renal cell carcinoma, immune checkpoint inhibitors, molecular biomarkers, prognosis, systemic therapy

## Abstract

Background/Objectives: Non-clear cell renal cell carcinoma (nccRCC) comprises biologically heterogeneous renal malignancies with distinct molecular drivers, clinical behavior, and therapeutic responses. This review summarizes prognosis and systemic therapy and evaluates emerging histology-specific and biomarker-directed approaches. Methods: We analyzed overall survival by histology in the SEER 17 Registries database (2000–2022) and conducted a narrative review of trials, guidelines, and translational studies. The ICD-O-3-defined population included 43,966 adults; 43,789 with known survival time were included in survival analyses. Results: Median follow-up was 96 months. Compared with papillary RCC, adjusted HRs were 0.67 (95% CI 0.64–0.70) for chromophobe RCC, 3.73 (3.37–4.11) for collecting duct carcinoma, 0.97 (0.80–1.17) for oncocytic tumors, and 33.23 (27.47–40.20) for medullary carcinoma. VEGFR- and MET-directed therapies and immune checkpoint inhibitor combinations show activity that varies by histology. Conclusions: nccRCC should be studied and managed as a collection of distinct entities. Registry associations require cautious interpretation because of residual confounding, evolving classification, and non-proportional hazards; subtype-specific trials and molecular stratification remain priorities.

## Simple Summary

Non-clear cell renal cell carcinomas (nccRCCs) comprise a heterogeneous group of kidney cancers with distinct biological features and clinical outcomes. Prognosis varies substantially according to histology and molecular drivers. Historically, outcomes have been inferior to those of clear cell RCC because subtype-specific evidence has been limited. Immune checkpoint inhibitors, targeted agents, and combination strategies have improved response rates, but optimal management remains uncertain. Progress will depend on molecular stratification, subtype-specific trials, and integration of genomic and epigenomic insights to individualize prognosis and therapy.

## 1. Introduction

Clear cell RCC (ccRCC), driven predominantly by alterations in the VHL pathway, constitutes most renal cell carcinomas and has well-established therapeutic paradigms. Non-clear cell renal cell carcinomas (nccRCCs) account for approximately 30% of renal malignancies [1]. Papillary RCC (pRCC), chromophobe RCC (chRCC), and tumors with mixed histology are among the more common forms, whereas collecting duct carcinoma, renal medullary carcinoma, oncocytic tumors, and other or unclassified tumors are rare and have distinct molecular drivers, cellular contexts, and clinical behavior [2].

Historically, patients with nccRCC have been underrepresented in prospective clinical trials, and management strategies have largely been extrapolated from studies conducted in ccRCC [3]. As a result, outcomes for patients with advanced nccRCC have generally been inferior, with lower response rates to conventional VEGF- and mTOR-targeted therapies and limited high-quality evidence to guide treatment selection. Moreover, the rarity of individual subtypes has posed challenges in conducting adequately powered, histology-specific studies, further contributing to gaps in evidence-based care.

Recent advances in molecular profiling and tumor biology have reshaped nccRCC as a collection of distinct diseases rather than a single clinical category [4]. These insights have identified actionable pathways, including MET alterations in papillary RCC, and have supported biomarker-driven and histology-enriched trials. Immune checkpoint inhibitors and combinations with tyrosine kinase inhibitors (TKIs) have shown promising activity in selected subtypes, although responses remain heterogeneous.

In this review, we summarize the prognostic landscape and systemic treatment options for nccRCC, emphasizing subtype-specific outcomes, emerging targeted and immunotherapeutic strategies, and the evolving role of molecular characterization. We also discuss ongoing trials and future directions for personalized management informed by tumor origin and biology.

## 2. Materials and Methods

We used a hybrid approach that combined a population-based SEER analysis with a narrative literature review to summarize demographic characteristics, survival outcomes, tumor biology, and therapeutic evidence in nccRCC.

We extracted de-identified data from the SEER Research Data, 17 Registries, November 2024 submission (diagnosis years 2000–2022) [2]. Eligible records were malignant kidney-parenchyma tumors (SEER site recode: Kidney Parenchyma) diagnosed at age ≥20 years and assigned to one of five prespecified ICD-O-3 histology groups: papillary (8260 and 8050), chromophobe (8317 and 8270), collecting duct (8319), oncocytic (8290 and 8342), or medullary (8510). Other renal parenchymal malignancies outside of the WHO 2022 classification were excluded. Patients younger than 20 years (n = 91) were excluded, yielding an adult cohort of 43,966 tumors.

Overall survival (OS) was defined as the interval from diagnosis to death from any cause or last follow-up. Patients with unknown survival time (n = 177) were excluded from survival analyses, leaving 43,789 evaluable patients. For 778 patients reported as surviving zero months, survival time was set to 0.5 months to retain early events.

Kaplan–Meier methods were used to estimate OS by histologic group, and differences were assessed using the log-rank test. Median follow-up was estimated with the reverse Kaplan–Meier method. Numbers at risk and deaths are reported with survival results.

Cox proportional hazards regression was used to estimate hazard ratios (HRs) and 95% confidence intervals (CIs), with papillary RCC as the reference group. Univariable models evaluated histology alone. Multivariable models adjusted for age, sex, race, and year of diagnosis. Stage-specific analyses were restricted to patients diagnosed during 2018–2022 with known Derived EOD 2018 stage because comparable stage data were unavailable for earlier years. The proportional-hazards assumption was assessed using Schoenfeld residuals. Because histology did not satisfy the assumption, Cox estimates are reported as average associations over follow-up and are interpreted together with Kaplan–Meier estimates at clinically relevant time points rather than as constant effects.

Analyses were performed using Stata MP 16.0 and independently reproduced for data verification. All tests were two-sided, and *p* < 0.05 was considered statistically significant.

The narrative review contextualized the population-based findings and summarized evidence on tumor biology, molecular drivers, biomarkers, and systemic therapy. PubMed/MEDLINE, major oncology journals, and ClinicalTrials.gov were searched through July 23, 2026, using combinations of “non-clear cell renal cell carcinoma,” “papillary,” “chromophobe,” “collecting duct,” “renal medullary,” “oncocytic,” “systemic therapy,” “clinical trial,” “molecular,” “transcriptomic,” and “epigenomics.” We prioritized peer-reviewed prospective phase II/III studies, contemporary clinical guidelines, subtype-specific translational studies, and registered ongoing interventional trials. Reference lists of included reports were also screened. Evidence was synthesized narratively because of substantial heterogeneity in histology, treatment, study design, and endpoints; cross-trial comparisons were considered indirect.

## 3. Results

Among 43,966 eligible tumor records in the ICD-O-3-defined cohort, papillary RCC was the predominant histology (n = 29,824; 67.83%), followed by chromophobe RCC (n = 13,297; 30.24%), collecting duct carcinoma (n = 511; 1.16%), oncocytic tumors (n = 208; 0.47%), and medullary carcinoma (n = 126; 0.29%) (Table 1).

Sex- and race-based distributions differed across histologic groups (Table 1). Papillary RCC comprised 74.02% of tumors among men and 53.09% among women and was particularly common among African American patients (81.23%). Chromophobe RCC comprised 44.82% of tumors among women compared with 24.13% among men. Medullary carcinoma represented 0.99% of tumors among African American patients but only 0.08% among White patients.

Among 43,789 patients with known survival time, 13,762 deaths occurred and 30,027 observations were censored alive. Median follow-up was 96 months. OS differed significantly by histology (global log-rank χ^2^ = 2914.51; df = 4; *p* < 0.001). Median OS was 229 months for chromophobe RCC, 161 months for papillary RCC, 131 months for oncocytic tumors, 16 months for collecting duct carcinoma, and 9 months for medullary carcinoma. With papillary RCC as the reference, univariable HRs were 0.57 (95% CI 0.55–0.60) for chromophobe RCC, 3.70 (95% CI 3.35–4.08) for collecting duct carcinoma, 1.29 (95% CI 1.07–1.56) for oncocytic tumors, and 11.42 (95% CI 9.46–13.78) for medullary carcinoma (Table 2).

**Figure 1:**
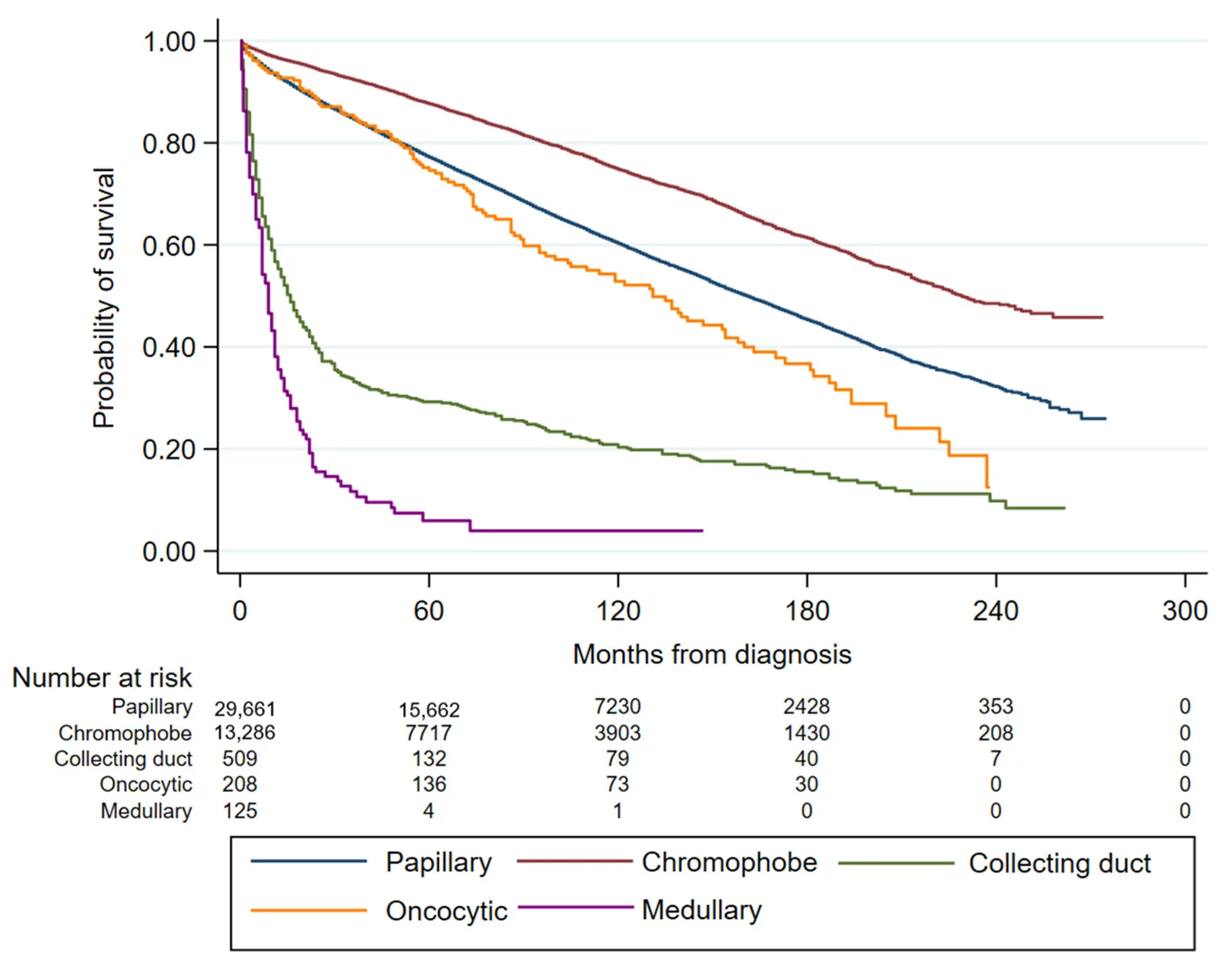
Kaplan–Meier estimates of overall survival for various nccRCC histologies. The risk table shows the number of patients remaining under observation.

After adjusting for age, sex, race, and diagnosis year, chromophobe RCC remained associated with better OS than papillary RCC (adjusted HR 0.67; 95% CI 0.64–0.70), whereas collecting duct carcinoma (adjusted HR 3.73; 95% CI 3.37–4.11) and medullary carcinoma (adjusted HR 33.23; 95% CI 27.47–40.19) remained associated with worse OS. Oncocytic histology was not independently associated with OS (adjusted HR 0.97; 95% CI 0.80–1.17; *p* = 0.743. Table 2 presents both models, including sample sizes, deaths, confidence intervals, and *p*-values. Figure 1 uses the same survival cohort. Stage-specific analyses included 9059, 1416, 1549, and 531 records in stages I–IV, respectively, and showed histology-related survival differences within each stage (all *p* < 0.001); median OS was 13 months for stage IV and was not reached for stages I–III. Schoenfeld-residual tests indicated non-proportional hazards for histology (including chromophobe and collecting duct carcinoma (both *p* < 0.001); consequently, the HRs should be interpreted as average associations over follow-up. Comparable stage, grade, metastatic-site, and treatment variables were not uniformly available across 2000–2022, precluding valid full-period adjustment for these factors.

### 3.1. Clinical Trials and Evolving Treatment Strategies

Non-clear cell renal cell carcinoma (nccRCC) represents a biologically heterogeneous group of malignancies comprising several histologic subtypes, each with distinct molecular and clinical characteristics [5,6]. Historically, therapeutic advances in this population have lagged behind those seen in clear cell RCC, largely due to the lower incidence of these tumors and their frequent exclusion from large, randomized trials. Consequently, treatment recommendations for nccRCC have often been extrapolated from studies conducted in clear cell RCC despite significant biological differences between these entities. Recently, however, there has been increasing recognition of the need for histology-specific investigation, leading to the development of prospective clinical trials dedicated to nccRCC.

Several prospective studies have helped define the current therapeutic landscape (Table 3 and Table 4). The randomized phase II ASPEN and ESPN trials compared VEGF-targeted therapy with mTOR inhibition and generally favored sunitinib over everolimus across most non-clear cell histologies, supporting VEGF-directed therapy as an initial approach [7,8]. Subsequent subtype-focused studies further refined treatment selection.

### 3.2. Papillary RCC

Papillary RCC has the strongest subtype-specific prospective evidence among nccRCCs. In PAPMET (SWOG-1500), cabozantinib improved progression-free survival and response rate compared with sunitinib [15]. SAVOIR suggested greater activity with savolitinib than with sunitinib in MET-driven papillary RCC, although the trial closed early [14]. KEYNOTE-427 Cohort B demonstrated activity with pembrolizumab monotherapy, particularly in papillary tumors [10]. Other studies reported encouraging results with cabozantinib plus nivolumab and with bevacizumab plus erlotinib in papillary and FH-deficient or HLRCC-associated disease; the latter regimen achieved an ORR of 72%, median PFS of 21.1 months, and median OS of 44.6 months in HLRCC-associated disease [11,13]. CALYPSO evaluated savolitinib combined with durvalumab and showed activity in the MET-driven subset [16].

ICI–TKI combinations have produced some of the strongest prospective activity reported in advanced nccRCC. In KEYNOTE-B61, pembrolizumab plus lenvatinib achieved an ORR of 49% in the primary report, while cabozantinib plus nivolumab achieved an ORR of 47.5% in the papillary, unclassified, and translocation-associated cohort [9,11]. Pembrolizumab monotherapy achieved an ORR of 26.7% in KEYNOTE-427 Cohort B [10]. In PAPMET, ORRs were 23% with cabozantinib and 4% with sunitinib in papillary RCC [15].

### 3.3. Chromophobe RCC

While papillary RCC appears to derive the greatest benefit from modern ICI-TKI combinations, chromophobe RCC has shown consistently lower activity with immunotherapy-based approaches. In KEYNOTE-427 Cohort B, ORRs were 28.8% for papillary RCC and 9.5% for chromophobe RCC; no responses were observed in the chromophobe cohort treated with cabozantinib plus nivolumab [10,11]. In ASPEN, chromophobe RCC was among the few settings in which outcomes numerically favored everolimus over sunitinib, supporting distinct biology and treatment sensitivity [8].

The low response rates reported for chromophobe RCC in KEYNOTE-427 and the cabozantinib–nivolumab study support a clinically distinct, relatively ICI-resistant phenotype [10,11].

### 3.4. Collecting Duct Carcinoma

The phase II BONSAI study evaluated cabozantinib in 23 patients with metastatic collecting duct carcinoma and reported an ORR of 35%, median PFS of 4 months, and median OS of 7 months. These results indicate measurable activity but do not establish comparative superiority because the study was small and single arm [18].

An emerging strategy is antibody-drug conjugate and immune checkpoint inhibitor combination therapy. The phase II REPRINT trial is evaluating pembrolizumab plus enfortumab vedotin in collecting duct carcinoma [27]. This histology-specific study is particularly important because the rarity of CDC has historically resulted in treatment recommendations derived from small prospective studies, retrospective series, and extrapolation from urothelial and other renal malignancies. For metastatic CDC, platinum-based chemotherapy remains an important treatment option, while cabozantinib represents a targeted therapeutic consideration and clinical trial enrollment should be prioritized whenever feasible. Current trials are evaluating combination regimens rather than targeting a single dominant molecular alteration.

Ongoing prospective studies reflect the shift toward histology-specific and immunotherapy–TKI strategies. Broad trials include the phase III STELLAR-304 study of zanzalintinib (XL092) plus nivolumab versus sunitinib and phase II studies of tivozanib plus nivolumab, cabozantinib plus nivolumab plus ipilimumab, and fruquintinib plus serplulimab [19,20,21,22]. Histology-enriched studies include PAPMET2 and the phase III SAMETA trial in MET-driven papillary RCC. REPRINT in collecting duct carcinoma further illustrates the move toward rare-subtype-specific trial design [23,24,25,27,28].

## 4. Discussion

### 4.1. Tumor Biology

**Figure 2:**
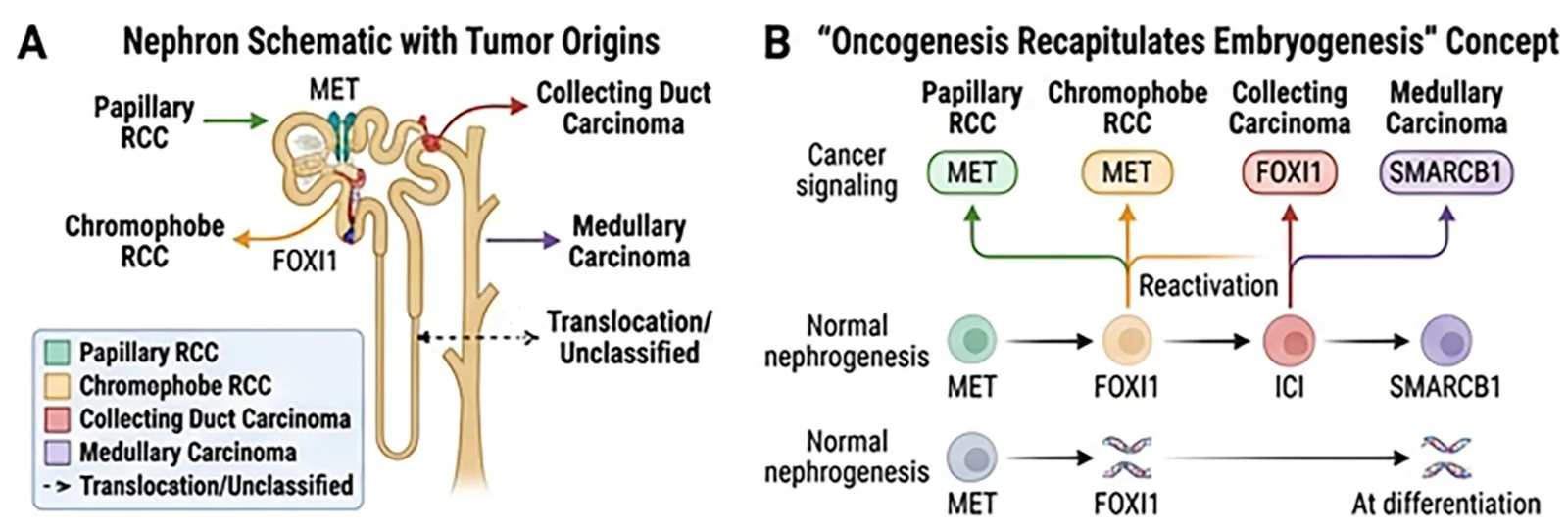
Proposed developmental context of major nccRCC subtypes. (**A**) Nephron segments associated with papillary, chromophobe, collecting duct, medullary, translocation-associated, and unclassified tumors. (**B**) Conceptual model in which oncogenic signaling reactivates lineage-associated developmental programs.

nccRCC subtypes demonstrate distinct molecular, histological, and clinical features compared to clear cell RCC, with limited effective therapeutic options and poor representation in clinical trials. Distinct RCC subtypes likely arise from progenitor cells located in different segments of the nephron, such as the proximal versus distal tubules, each retaining unique transcriptional and epigenetic programs established during kidney development (Figure 2A). In this context, identical or overlapping genetic alterations may produce divergent phenotypes depending on the cellular context in which they occur (Figure 2B). This paradigm aligns with a stem cell-based model of oncogenesis, wherein tumor heterogeneity reflects the differentiation capacity and plasticity of the cell-of-origin. Consequently, biomarkers based solely on genomic alterations may be insufficient, as they fail to capture the hierarchical organization and stem-like properties of tumors. Instead, integrating developmental lineage, cellular differentiation state, and microenvironmental context may improve diagnostic precision, prognostication, and therapeutic targeting. Such a framework has important implications for translational research, emphasizing the need to move beyond reductionist genomic approaches toward integrated, context-dependent models of cancer biology.

#### 4.1.1. Developmental and Embryonic Origins

The kidney develops through reciprocal interactions between the metanephric mesenchyme and ureteric bud, which generate nephron epithelia and the collecting system, respectively [29]. Single-cell transcriptomic and chromatin-accessibility (scATAC-seq) data support a model in which major nccRCC subtypes arise within distinct nephron-segment or progenitor contexts, with tumor heterogeneity reflecting both developmental lineage and subsequent oncogenic and metabolic reprogramming. Most papillary RCCs show proximal-tubule programs, although subsets may reflect collecting-duct principal cells or renal tubular progenitors that expand in chronic renal insufficiency or in association with papillary adenomas [30,31,32,33]. Chromophobe RCC is linked to distal nephron and collecting-duct intercalated-cell programs, supported by gene-expression, methylation, and immunohistochemical markers including KIT (CD117), CK7, and FOXI1. Collecting duct carcinoma also shows distal nephron or collecting-duct features [34,35]. Renal medullary carcinoma is thought to arise in the medullary epithelium under chronic hypoxic stress.

Several molecular programs that characterize nccRCC—including MET signaling in pRCC, FOXI1-driven transcription in chRCC, and Wnt/β-catenin activity across subtypes—may represent reactivation of developmental pathways rather than entirely de novo programs acquired during malignant transformation. This concept, in which oncogenesis partially recapitulates embryogenesis in an inappropriate cellular context, may explain lineage-associated phenotypes and therapeutic vulnerabilities [36].

During kidney development, nephron progenitors acquire spatially and temporally regulated identities rather than following a simple fixed hierarchy. A stem-cell framework therefore suggests that tumor phenotypes may reflect both the developmental context of the initiating cell and subsequent differentiation or dedifferentiation. This hypothesis may help explain why some distal-nephron-associated tumors are aggressive, whereas a highly differentiated tumor such as chromophobe RCC may remain relatively indolent. These relationships remain conceptual and should not be interpreted as proof that nephron position alone determines clinical behavior.

#### 4.1.2. Heterogeneity Across nccRCC Subtypes

Heterogeneity in nccRCC occurs at several levels. Intertumoral variation reflects distinct developmental and molecular contexts and may be further shaped by stem-like plasticity, such that similar genetic or epigenetic alterations produce different phenotypes depending on the cellular state in which they occur [33]. Spatial and temporal diversity within tumors also permits resistant subclones to emerge during treatment.

Transcriptomic studies in ccRCC provide proof of principle for classifying renal tumors by functional state rather than morphology alone. Verbiest et al. described favorable angiogenic or normal-like groups (ccrcc2/classical, approximately 45%; ccrcc3/normal-like, approximately 5%) and less favorable, less differentiated groups (ccrcc1/c-MYC-up, approximately 30%; ccrcc4/c-MYC-up and immune-up, approximately 20%). The ccrcc4 phenotype was enriched for inflammatory and sarcomatoid features and expressed an immunosuppressive program. Although these ccRCC classifiers cannot be applied directly to nccRCC, the same principles—transcriptomic state, immune contexture, methylation pattern, and differentiation status—may help explain divergent behavior and treatment response among tumors with similar morphology or overlapping mutations [37].

In the TRACERx Renal model, multiregion sequencing of ccRCC identified seven recurrent evolutionary subtypes and linked metastatic behavior to intratumoral heterogeneity (ITH) and somatic copy-number alteration (SCNA) burden [38,39]. Tumors with low ITH but high SCNA burden were associated with rapid multisite progression and included multiple-clonal-driver, VHL-wild-type, and BAP1-driven trajectories. Highly branched, high-ITH tumors, often involving PBRM1-led trajectories, tended to progress more slowly, whereas low-ITH, low-SCNA tumors had relatively low metastatic potential. Metastases were more clonally homogeneous than matched primary tumors, consistent with a metastatic bottleneck. These evolutionary classes are biological and prognostic frameworks, not validated treatment-selection rules, and their application to nccRCC requires histology-specific multiregion cohorts and prospective validation.

Sarcomatoid dedifferentiation can arise in any RCC histology and exemplifies an extreme epithelial-to-mesenchymal transition, with loss of epithelial identity, acquisition of invasive and stem-like properties, treatment resistance, and paradoxically greater sensitivity to immune checkpoint inhibition [36,40]. Sarcomatoid change is therefore best interpreted as a dedifferentiation pattern rather than a distinct histology.

The tumor microenvironment further contributes to heterogeneity by shaping immune infiltration, angiogenesis, and stromal interactions. Notably, single-cell analyses suggest that tumor epithelial cells actively recruit immune cells, which may partly explain the sensitivity of some RCCs to immune checkpoint inhibitors despite relatively low neo-antigen burden [31].

Papillary RCC is associated with MET alterations, particularly in tumors previously classified as type 1, whereas CDKN2A loss, SETD2 mutations, and NRF2–ARE pathway activation are associated with less favorable biology [41,42]. TP53 and PTEN mutations occur in chromophobe RCC, which otherwise has a low somatic mutation burden and a distinct oncogenic process. Collecting duct and renal medullary carcinomas are rare, aggressive tumors; renal medullary carcinoma is characterized by SMARCB1 (INI-1) loss and is strongly associated with sickle cell trait [1].

#### 4.1.3. Translational and Therapeutic Implications

Cancer treatment has increasingly shifted from grouping tumors solely by histomorphology toward integrating developmental lineage and molecular context, although their clinical utility in nccRCC remains unproven [43]. Treatment selection should therefore remain grounded in histology-specific clinical evidence and validated molecular findings.

In papillary RCC, germline or somatic MET pathway activation represents one of the most actionable molecular alterations. This rationale supports MET-directed agents, including cabozantinib and savolitinib, particularly in molecularly selected patients. Transcriptome-derived stemness indices in pRCC have been associated with stage and histologic phenotype; tumors previously classified as type 2 showed higher stemness scores and worse outcomes than type 1 tumors, suggesting that dedifferentiation may track with clinical aggressiveness [31].

In chromophobe RCC, intercalated-cell lineage programs and FOXI1-associated epigenomic features distinguish the tumor from pRCC. Low mutation burden, limited neoantigen load, and T-cell exclusion may contribute to reduced responsiveness to immune checkpoint inhibition [44]. In ccRCC models, SNX5-mediated CD44 internalization reduced stemness-associated and EMT phenotypes; whether this mechanism influences immune exclusion or ICI response in nccRCC is unknown [45]. PTEN loss, chromosomal alterations, and mTOR-pathway activity may help explain the modest activity observed with everolimus in the chromophobe subgroup of ASPEN.

Collecting duct and renal medullary carcinomas are aggressive distal-nephron-associated tumors in which tumor-suppressor loss and epigenomic dysregulation may be more important than a single dominant oncogenic driver. Their plasticity and molecular complexity suggest that combinations targeting multiple compartments may be required. In collecting duct carcinoma, co-occurring CDKN2A loss and SLC7A11 overexpression have been associated with cell-cycle dysregulation and potential cisplatin resistance [46].

This transition from histomorphology-based classification to biology-informed treatment selection supports a broader focus on therapy development: determining how, when, and in whom to deploy existing agents, in addition to developing new drugs [43].

#### 4.1.4. Biomarkers

Genomic and epigenomic biomarkers should be distinguished when interpreting nccRCC biology (Table 5). TP53 alterations can occur in both chromophobe RCC, which had favorable survival in our cohort (univariable HR 0.57), and collecting duct carcinoma, which had poor survival (univariable HR 3.70), demonstrating that mutation status alone does not determine phenotype. Lineage-associated transcriptional programs, including FOXI1 in chRCC and HNF1B in pRCC, and subtype-specific methylation patterns may add prognostic information beyond mutation burden. Cellular context and epigenomic state should therefore be integrated with genomic alterations [40].

### 4.2. Future Directions

Future progress in nccRCC will require moving beyond treatment as a single clinical entity toward histology-specific and biologically informed strategies. Papillary, chromophobe, collecting duct, medullary, oncocytic, translocation-associated, and other or unclassified tumors harbor distinct molecular features that influence behavior and treatment response [47]. Subtype-enriched and biomarker-informed trials are needed to evaluate these biologically distinct tumors. A refined understanding of tumor pathophysiology may improve the use of existing drugs as much as the identification of new targets. For example, preclinical and early clinical observations with panitumumab in SMARCB1-deficient renal medullary carcinoma support EGFR dependence as a potential vulnerability and justify further evaluation of EGFR-directed antibodies [48].

At the same time, accumulating experimental evidence suggests that intratumoral heterogeneity and therapeutic resistance in renal cancers may be partly sustained by tumor cell populations with stem-like properties [49]. Several putative stem cell-associated markers, including CD133, CD44, and CXCR4, have been described in RCC and have been associated with stem-like behavior, tumorigenicity, and adverse clinical features in preclinical and translational studies [50]. However, their precise biologic and clinical significance, particularly across individual nccRCC subtypes, remains incompletely defined.

An important area for future study involves the development of adaptive and biologically stratified clinical trials. Given the rarity of many nccRCC subtypes, basket or platform trial designs that incorporate molecular stratification may accelerate the evaluation of targeted therapies and combination regimens in defined patient subsets. Finally, integrating longitudinal sampling strategies, such as circulating tumor DNA or serial tumor biopsies, may provide insight into clonal evolution and mechanisms of therapeutic resistance over time [51]. Epigenomic characterization of ctDNA, including methylation-based assays, warrants prospective evaluation for longitudinal monitoring in nccRCC. Whether these assays identify stem-like residual cell states more effectively than mutation-based assays has not been established.

ctDNA may complement lineage- and tissue-based biomarkers as a dynamic marker of molecular residual disease and treatment response. In colorectal cancer, longitudinal methylation-based ctDNA testing has demonstrated strong associations between postoperative or post-adjuvant positivity and recurrence risk [52]. Although ctDNA is not specific to cancer stem cells, methylation and other epigenomic features may detect clinically relevant residual cell states that mutation-only assays do not fully capture.

In RCC, tumor-informed ctDNA positivity has been associated with recurrence in localized disease and with inferior progression-free survival in advanced disease. Pretreatment positivity was associated with shorter PFS (HR 5.51; *p* = 0.008), and positivity at 3 months was associated with worse PFS (HR 18.42; *p* = 0.007); favorable ctDNA dynamics corresponded to a longer median time to progression (10.3 vs. 1.7 months) [53]. In a separate post-nephrectomy cohort of 77 patients, including 13 with nccRCC, molecular residual disease positivity was associated with inferior recurrence-free survival (HR 18; *p* < 0.0001) [54]. Small studies also suggest that ctDNA can precede radiographic progression, clarify equivocal imaging, and track response or clearance after resection of recurrent RCC [55,56].

However, RCC is often a low-shedding tumor, and the available evidence is dominated by clear cell disease, retrospective cohorts, conference reports, and small case series. A positive ctDNA result may complement imaging, but a negative result cannot exclude residual disease. Cost, assay sensitivity, and limited negative predictive value also constrain routine use. Prospective, histology-specific studies are required before methylation- or other epigenomic-based ctDNA assays can guide therapy or serve as a validated biomarker of stemness.

### 4.3. Limitations

This study has several limitations. First, the SEER analysis was retrospective and relied on registry-recorded demographic, pathological, and survival data. Consequently, the observed associations cannot establish causality and may be affected by residual confounding, incomplete data, coding errors, and unmeasured differences in comorbidities, performance status, treatment selection, and access to care. SEER records tumors rather than unique individuals; therefore, an eligible tumor was retained when a patient had multiple primary malignancies. In addition, overall survival includes deaths from both cancer-related and noncancer causes and may be influenced by differences in age and comorbidity across histologic groups.

Second, histologic groups were defined using prespecified ICD-O-3 codes. Because the 2022 WHO classification includes molecularly defined entities and SEER does not provide comprehensive molecular characterization, the registry-based categories do not fully correspond to contemporary WHO entities.

Third, comparable stage, pathological grade, metastatic-status, and treatment variables were not uniformly available throughout the study period. Stage-, grade-, and M-stage analyses were consequently restricted primarily to patients diagnosed from 2018 onward. Information regarding systemic therapy, treatment sequence, drug exposure, response, recurrence, comorbidity, and performance status was unavailable. Although the multivariable models adjusted for age, sex, race, and year of diagnosis, full-period adjustment for stage, grade, metastatic burden, surgery, and systemic treatment was not possible. Age adjustment used grouped-age approximations, including an assumed age of 92 years for the top-coded 90+ category.

Finally, collecting duct, oncocytic, and medullary tumors were uncommon, producing sparse data and imprecise estimates in subgroup analyses. Some subgroup estimates could not be calculated reliably and should be considered exploratory.

## 5. Conclusions

nccRCC comprises biologically heterogeneous malignancies with marked prognostic diversity. In our ICD-O-3-defined SEER cohort, chromophobe RCC had the most favorable survival, whereas collecting duct and medullary carcinomas had the poorest outcomes. These differences are consistent with distinct developmental contexts, epigenomic programs, and degrees of cellular plasticity across subtypes. Similar or overlapping somatic alterations may therefore produce different clinical phenotypes depending on lineage and epigenomic state [36,40].

**Figure 3:**
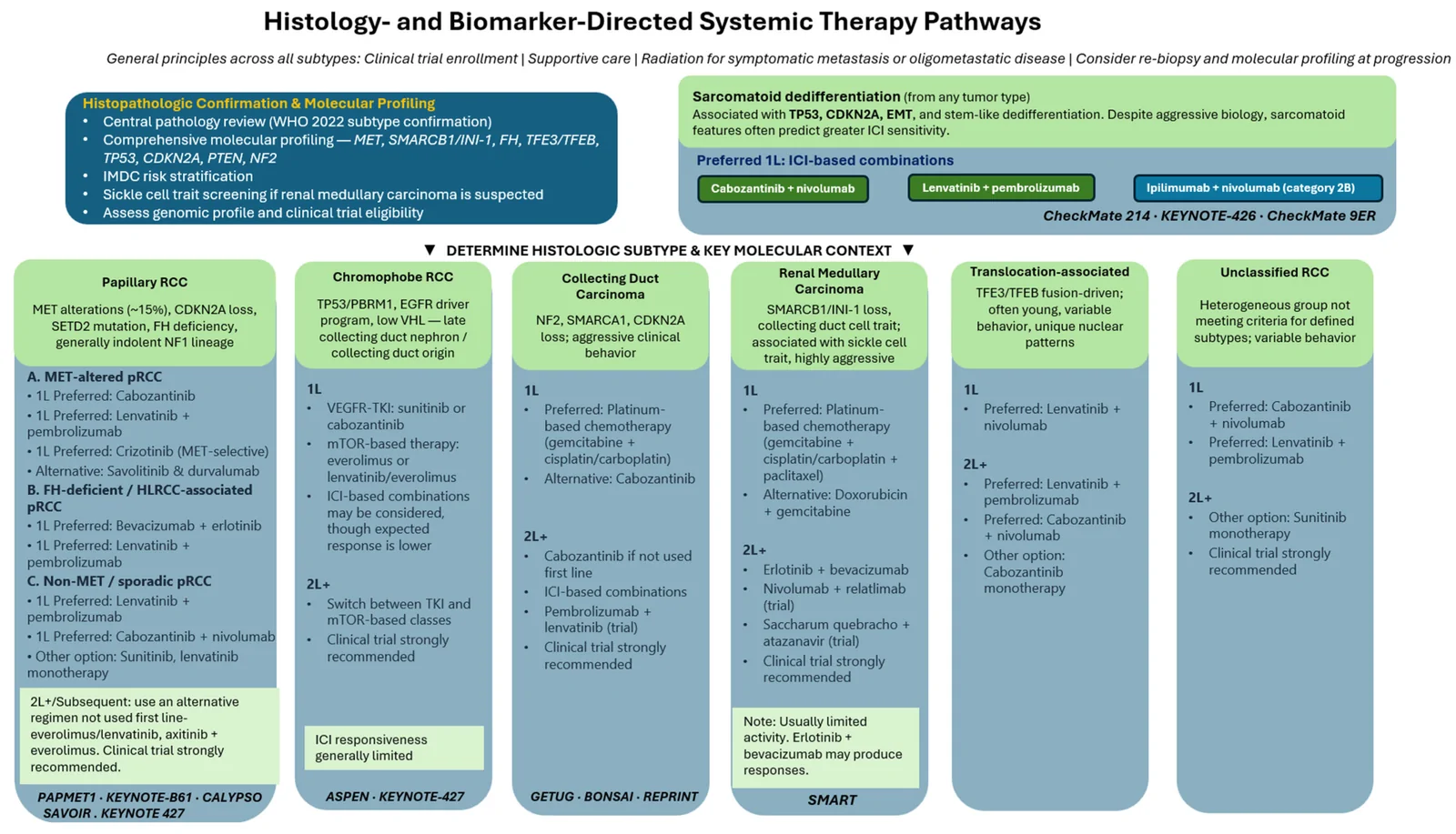
Histology- and biomarker-directed systemic therapy pathways for advanced nccRCC. Recommendations should be individualized according to histology, molecular context, prior therapy, comorbidity, guideline availability, and clinical-trial eligibility.

Prospective nccRCC trials likewise demonstrate heterogeneous treatment activity and support therapy development that identifies the appropriate agents, combinations, sequences, and patient populations (Figure 3) [43]. Integrating molecular profiling, histology-enriched trial design, and biomarker-informed patient selection offers the most promising path toward improved outcomes in these rare malignancies.

## Figures and Tables

**Table 1:** Distribution of various nccRCC histologies overall and by sex, age, race and stage IV.

Histological Type	Total, n (%)	Sex		Median Age, Years	Race			Stage IV, n/N Known (%)
		Male, n (%)	Female, n (%)		White, n (%)	African American, n (%)	Other, n (%)	
Papillary	29,824 (67.83)	22,924 (74.02)	6900 (53.09)	62	20,425 (64.44)	8102 (81.23)	1297 (56.46)	388/8467 (4.6)
Chromophobe	13,297 (30.24)	7472 (24.13)	5825 (44.82)	62	10,721 (33.83)	1628 (16.32)	948 (41.27)	82/3993 (2.1)
Collecting duct	511 (1.16)	354 (1.14)	157 (1.21)	62	361 (1.14)	111 (1.11)	39 (1.70)	30/66 (45.5)
Oncocytic	208 (0.47)	130 (0.42)	78 (0.60)	72	164 (0.52)	34 (0.34)	10 (0.44)	NE (0 known)
Medullary	126 (0.29)	90 (0.29)	36 (0.28)	30	24 (0.08)	99 (0.99)	3 (0.13)	33/40 (82.5)

Percentages in the sex and race columns use the corresponding column total as the denominator. Other race includes Asian or Pacific Islander, American Indian/Alaska Native, and unknown race. Median ages are approximated from age groups and rounded to whole years. Stage IV values are restricted to diagnoses during 2018–2022 with known stage I–IV; NE, not estimable.

**Table 2:** Univariable and multivariable Cox regression results for overall survival by histology.

Histological Type	N	Deaths	Univariable HR	Univariable 95% CI	Univariable *p*-Value	Multivariable HR	Multivariable 95% CI	Adjusted *p*-Value
Papillary	29,661	10,221	1.00	Reference	—	1.00	Reference	—
Chromophobe	13,286	2904	0.57	0.55–0.60	<0.001	0.67	0.64–0.70	<0.001
Collecting duct	509	413	3.70	3.35–4.08	<0.001	3.73	3.37–4.11	<0.001
Oncocytic	208	113	1.29	1.07–1.56	0.007	0.97	0.80–1.17	0.743
Medullary	125	111	11.42	9.46–13.78	<0.001	33.23	27.47–40.19	<0.001

HR, hazard ratio; CI, confidence interval; Univ., univariable; adjusted, multivariable. Both models include 43,789 records and 13,762 deaths, with papillary RCC as the reference and Breslow handling of tied event times. The multivariable model adjusts for approximate age and diagnosis year as continuous variables, sex, and five race categories, including unknown race. *p*-values are two-sided Wald tests. Non-proportional hazards were detected; HRs summarize average associations over follow-up.

**Table 3:** Completed prospective trials in advanced nccRCC. Completed trials.

Name of Trial	Phase	Population	Intervention	Comparator	Key Points
KEYNOTE-B61 (NCT04704219) [9]	II	Advanced nccRCC (*n* = 158)	Pembrolizumab + lenvatinib	Single arm	ORR 49% (78/158) in the primary report; activity varied by histology
KEYNOTE-427 (NCT02853344) [10]	II	Advanced nccRCC (*n* = 165)	Pembrolizumab	Single arm	ORR 26.7%; activity varied by histology
NCT03635892 [11]	II	Advanced nccRCC (*n* = 47)	Cabozantinib + nivolumab	Single arm	ORR 47.5% in the papillary, unclassified, and translocation-associated cohort; no responses in the chromophobe cohort
ASPEN (NCT01108445) [8]	II	Metastatic nccRCC (*n* = 108)	Sunitinib	Everolimus	Median PFS 8.3 months with sunitinib versus 5.6 months with everolimus; interpretation by histology was exploratory
SUNNIFORECAST (NCT03075423) [12]	II	Advanced nccRCC (*n* = 299)	Ipilimumab + nivolumab	Standard of care (TKI, TKI/IO, other)	Median PFS: 5.4 months with ipilimumab plus nivolumab versus 5.7 months with standard of care; 12-month OS: 78% versus 68%, respectively
NCT01130519 [13]	II	HLRCC-associated or sporadic papillary RCC (*n* = 83)	Bevacizumab + erlotinib	Single arm	HLRCC: ORR 72%, median PFS 21.1 months, median OS 44.6 months; sporadic pRCC: ORR 35%, median PFS 8.9 months
SAVOIR (NCT03091192) [14]	III	MET-driven papillary RCC (*n* = 60)	Savolitinib	Sunitinib	ORR 27% with savolitinib versus 7% with sunitinib; trial closed early and was underpowered for definitive comparison
PAPMET/SWOG-1500 (NCT02761057) [15]	II	Metastatic papillary RCC (*n* = 147)	Cabozantinib	Sunitinib	Cabozantinib improved median PFS (9.0 vs. 5.6 months) and ORR (23% vs. 4%) versus sunitinib
CALYPSO (NCT02819596) [16]	II	Advanced papillary RCC (*n* = 41)	Savolitinib + durvalumab	Single arm	ORR 29%; median PFS 4.9 months with savolitinib plus durvalumab
NCT01399918 [17]	II	Advanced pRCC or nccRCC with papillary features (*n* = 35)	Everolimus + bevacizumab	Single arm	ORR 29%; median PFS 11.0 months with everolimus plus bevacizumab
BONSAI (NCT03354884) [18]	II	Metastatic collecting duct carcinoma (*n* = 23)	Cabozantinib	Single arm	ORR 35%; median PFS 4 months and median OS 7 months with cabozantinib

pRCC: Papillary renal cell carcinoma; uRCC: unclassified renal cell carcinoma.

**Table 4:** Selected ongoing or recently active clinical trials in nccRCC. Ongoing trials.

Name of Trial	Phase	Population	Intervention	Comparator Arm	Status
STELLAR-304 (NCT05678673) [19]	III	Advanced/metastatic non-clear cell RCC	Zanzalintinib + Nivolumab	Sunitinib	Recruiting
NCT06053658 [20]	II	Advanced nccRCC	Tivozanib + Nivolumab	Single arm	Recruiting
NCT04413123 [21]	II	Advanced nccRCC	Cabozantinib + Nivolumab + Ipilimumab	Single arm	Not currently recruiting
NCT05831891 [22]	II	Advanced nccRCC	Fruquintinib + Serplulimab	Single arm	Recruiting
PAPMET2/S2200 (NCT05411081) [23]	II	Metastatic papillary RCC	Cabozantinib + Atezolizumab	Cabozantinib	Recruiting
SAMETA (NCT05043090) [24]	III	MET-driven papillary RCC	Savolitinib + Durvalumab	Sunitinib or Durvalumab	Not currently recruiting
NCT05096390 [25]	II	Papillary RCC	Axitinib + Pembrolizumab	Axitinib	Recruiting
NCT04981509 [26]	II	Papillary RCC	Bevacizumab + Erlotinib + Atezolizumab	Single arm	Not currently recruiting
REPRINT (NCT06302569) [27]	II	Collecting duct carcinoma	Pembrolizumab + Enfortumab vedotin	Single arm	Recruiting
NCT05808608 [28]	I	Sarcomatoid or rare RCC subtypes (including nccRCC)	Cadonilimab * + Axitinib	Single arm	Recruiting

* AK104 is a PD-1/CTLA-4 bispecific antibody.

**Table 5:** Developmental context, molecular alterations, transcriptomic/epigenomic biomarkers, and biological features of selected nccRCC subtypes and related patterns.

Subtype	Proposed Cell or Segment of Origin	Key Molecular Alterations	Transcriptomic/Epigenomic Markers and Pathways	Biological Features
Papillary	PCT with subset from CD	MET (~15%), CDKN2A loss, SETD2, FH	HNF1B lineage program; MET signaling; C1 epigenomic cluster	Heterogeneous
Chromophobe	DCT/CD	TP53, PTEN	FOXI1 intercalated-cell program; KIT/CK7; C2 epigenomic cluster	Generally indolent
Collecting duct	Distal nephron/collecting duct	NF2, SMARCB1	Distal-nephron program; NF2/SMARCB1-associated and methylation abnormalities	Aggressive
Renal Medullary	Medullary CD	SMARCB1 (INI-1) loss	SMARCB1-loss program; C2 epigenomic cluster	Highly aggressive (classically associated with sickle cell trait)
Sarcomatoid	Dedifferentiation from any RCC subtype	TP53, CDKN2A, others (heterogeneous)	Variable	Highly aggressive, poor prognosis
Translocation RCC	Variable	TFE3/TFEB gene fusions	C1 epi-cluster, ACTG1-MITF fusion	Younger patients
Unclassified or emerging	Variable	TSC2/MTOR, ALK, FH	Variable	Heterogeneous

Proposed developmental contexts and molecular associations are not validated treatment-selection rules. Sarcomatoid change is a dedifferentiation pattern that can occur in any RCC subtype. PCT, proximal convoluted tubule; DCT, distal convoluted tubule; CD, collecting duct.

## Data Availability

The data analyzed in this study are available from the National Cancer Institute SEER Program after execution of the SEER Research Data Use Agreement (SEER 17 Registries Research Data, November 2024 submission, 2000–2022). The ICD-O-3 selection criteria and analytic methods are reported in the manuscript.

## Abbreviations

The following abbreviations are used in this manuscript:

ALK	Anaplastic lymphoma kinase
ccRCC	Clear cell renal cell carcinoma
CD	Collecting duct
CDC	Collecting duct carcinoma
CDKN2A	Cyclin-dependent kinase inhibitor 2A
chRCC	Chromophobe renal cell carcinoma
CI	Confidence interval
CK7	Cytokeratin 7
CSC	Cancer stem cell
CTLA-4	Cytotoxic T-lymphocyte-associated protein 4
ctDNA	Circulating tumor DNA
CXCR4	C-X-C chemokine receptor type 4
DCT	Distal convoluted tubule
EMT	Epithelial-to-mesenchymal transition
FH	Fumarate hydratase
FOXI1	Forkhead box I1
HLRCC	Hereditary leiomyomatosis and renal cell carcinoma
HNF1B	Hepatocyte nuclear factor 1 beta
ICI	Immune checkpoint inhibitor
INI-1	Integrase interactor 1
KIT/CD117	KIT proto-oncogene receptor tyrosine kinase/cluster of differentiation 117
MITF	Microphthalmia-associated transcription factor
mTOR	Mechanistic target of rapamycin
MTF	Master transcription factor
nccRCC	Non-clear cell renal cell carcinoma
NF2	Merlin
NRF2-ARE	Nuclear factor erythroid 2-related factor 2–antioxidant response element
PCT	Proximal convoluted tubule
PD-1	Programmed death-1
PFS	Progression-free survival
pRCC	Papillary renal cell carcinoma
scATAC-seq	Single-cell assay for transposase-accessible chromatin sequencing
SETD2	SET domain-containing 2
SLC7A11	Solute carrier family 7 member 11
SMARCB1	SWI/SNF-related matrix-associated actin-dependent regulator of chromatin subfamily B member 1
TFE3	Transcription factor E3
TFEB	Transcription factor EB
TGF-beta	Transforming growth factor beta
TKI	Tyrosine kinase inhibitor
TSC2	Tuberous sclerosis complex 2
uRCC	Unclassified renal cell carcinoma
VEGF	Vascular endothelial growth factor
VEGFR	Vascular endothelial growth factor receptor
VHL	Von Hippel-Lindau
